# Maternal feeding styles in infancy predict child body mass index z-scores at 72 months: findings from a cohort of Jamaican children

**DOI:** 10.3389/fpubh.2023.1161808

**Published:** 2023-06-30

**Authors:** Amika S. Wright, Natalie Guthrie-Dixon, Marshall K. Tulloch-Reid, Susan M. Chang, Susan P. Walker

**Affiliations:** Epidemiology Research Unit, Caribbean Institute for Health Research, The University of the West Indies, Mona, Jamaica

**Keywords:** feeding styles, restrictive, obesity, overweight, Jamaica

## Abstract

**Objective:**

To explore whether maternal feeding styles at 12 months predict child Body Mass Index (BMI) z-scores at 72 months and evaluate whether BMI z-scores at 18 months mediates the association.

**Methods:**

Data from 239 mother–child pairs participating in a parenting intervention were analyzed. Feeding information was ascertained at 12 months by questionnaire with feeding styles identified using factor analysis. Children’s weight and length/height were measured at 18 and 72 months and BMI z-scores computed. Maternal sociodemographic data, depressive symptoms and language skills were obtained by questionnaire. Multilevel linear regression analysis was used to examine whether feeding styles predicted 72 month BMI z-scores. Complete case analysis was conducted and multiple imputation used to treat missing values of explanatory and outcome variables at 18 and 72 months. Mediational analysis was performed to assess the indirect effects of feeding styles on 72 month BMI z-scores via 18 month BMI z-scores.

**Results:**

Restrictive feeding at age 12 months predicted higher 72 month BMI z-scores in both complete case analysis [*β* (95%CI): 0.19 (0.02, 0.37)] and multiple imputation [*β* (95%CI): 0.20 (0.02, 0.39)]. Uninvolved, forceful, indulgent and responsive feeding styles were not significant predictors of 72 month BMI z-scores. A significant indirect effect was observed between restrictive feeding and child BMI z-scores at 72 months via 18 month BMI z-scores [*β* (95% CI) 0.12 (0.03, 0.22)].

**Conclusion:**

Restrictive feeding at 12 months was associated with higher BMI z-scores at 72 months and appeared to be mediated by BMI z-scores at 18 months.

## 1. Introduction

The global prevalence of overweight and obesity in adults has been steadily increasing (1); reducing quality of life and resulting in adverse health outcomes including type 2 diabetes, coronary heart disease, asthma, and arthritis (2). Data from National Surveys in the Caribbean, Latin America, North and South Africa, the Middle East and the Pacific Islands report obesity as an important health concern (1). Childhood overweight and obesity prevalence has also increased and was estimated to affect 39 million children globally under 5 years of age, with approximately 4 million children in Latin America and the Caribbean living with overweight in 2020 (3, 4). Children with overweight are at greater risk of adult overweight/obesity with the influence of childhood overweight/obesity on adult status increasing as children become older (5, 6). Across 10 Caribbean territories (Grenada, Dominica, Trinidad and Tobago, Jamaica, St. Kitts and Nevis, Antigua, St. Vincent and the Grenadines, Barbados, Belize, St. Lucia) the prevalence estimates of school-aged children (6–12 years) in 2018 ranged from 28.0–44.5% for overweight and 14.3–19.8% for obesity (7). In Jamaica, overweight prevalence in 2018 for 5 year old children was 5.9% (8).

Childhood overweight and obesity may be attributed to several factors including genetics, the built environment, physical inactivity, parent behavior and feeding styles (9). Parent feeding styles are of significance as they are potentially modifiable risk factors for overweight and obesity in infancy and early childhood and may affect later weight outcomes through influence on child eating behavior. Feeding styles have been the target of interventions aimed at preventing overweight and obesity during infancy (10–12). There is growing evidence to suggest that non-responsive feeding styles (uninvolved, restrictive, forceful and indulgent) may promote childhood overweight and obesity by hindering the child’s development of self-regulation skills (13, 14). These feeding styles may increase a young child’s risk of overweight/obesity by (1) allowing them increased access to calorie dense foods (13–15); (2) controlling the feeding environment with little sensitivity to the child’s internal hunger and satiety cues (13, 14); or (3) allowing the child to determine their nutritional intake with a lack of parental control (13, 14).

Longitudinal studies examining the associations between non-responsive feeding styles and child adiposity have revealed mixed findings (14, 16–21). Faith et al. reported that restrictive feeding at age 5 years was associated with higher body mass index (BMI) z-scores at age 7 years (17) while Francis and Birch reported that overweight mothers who practiced more restrictive feeding had daughters who showed greater increases in eating in the absence of hunger between the ages of 5–9 years and subsequently higher BMI (22). Campbell et al. explored the relationship between restrictive feeding and change in BMI 3 years later among 5–6 year olds and 10–12 year olds (16). There was no significant association between baseline feeding restriction and baseline BMI in either age group, however, at the 3-year follow-up, restrictive feeding predicted lower BMI in 5–6 year olds, but there was no association in 10–12 year olds (16). A study by Rifas-Shiman et al. showed restrictive feeding at age 1 year was associated with higher BMI z-scores at age 3 years but not after adjusting for baseline BMI z-scores (19). Other longitudinal studies have stated varied findings of either no association (21) or an inverse association (18) between forceful feeding and child BMI.

The association between feeding style and child weight is thought to be directed from parent to child with feeding styles influencing child weight status (23, 24). Notwithstanding, it is also probable that characteristics of the child, such as weight status may influence parental feeding styles (20, 21, 25). Observational and theoretical studies have suggested that the relationship between child weight and parental feeding styles may be influenced by factors such as parental perception of child weight status and concern about future risk of overweight, actual child weight, child problematic eating behavior, child diet, parental weight status and parental body dissatisfaction (22, 25–27). Webber et al. reported that, among children aged 7–9 years, maternal overweight concern mediated the association between restrictive feeding and child weight (25). Findings from Birch and colleagues have shown that higher child weight status was associated with increased parental restrictive feeding suggesting use of restrictive feeding may be greater in children affected by overweight or obesity than non-affected children (28, 29). A study by Grimmett et al. revealed an increase in restrictive feeding by parents of 6–11 year olds following feedback about their child’s overweight status (30). Parents who were told their child was overweight restricted their child’s food intake as an overweight reduction mechanism (30). Parental concern about the child’s current or future weight status may be another mechanism linking restrictive feeding to child weight (25, 29, 31, 32).

Previous studies have examined the association between feeding styles and child BMI in mostly high-income countries, with only a few conducted in low- and middle-income countries (LMICs) (14, 16–22). The aim of this study was to examine the longitudinal association between maternal feeding styles at 12 months and child BMI z-scores at 72 months in a cohort of Jamaican children and to evaluate using a mediational model whether any effect of feeding styles on child BMI z-scores at 72 months was through the influence of the child’s BMI z-scores at age 18 months. We hypothesized that maternal feeding styles during infancy would be associated with child BMI z-scores at 72 months and that non-responsive feeding styles would be associated with higher BMI z-scores at 72 months. We also explored whether any association would be mediated by 18 month BMI z-scores.

## 2. Materials and methods

### 2.1. Study participants

This study was a longitudinal assessment that examined the association between maternal feeding styles and later childhood overweight. Mothers and children were participants in a cluster randomized trial of a parenting intervention designed to improve mother child interactions and promote early childhood stimulation in Jamaica, Antigua and St. Lucia when children were 3 to 18 months of age (33). Mother–child dyads were recruited at the 6–8 week postnatal clinic from 39 health centers in Jamaica (*n* = 20), Antigua (*n* = 10), and St. Lucia (*n* = 9). A total of 601 mother–child pairs were enrolled in the parenting trial: 396 from Jamaica, 102 from Antigua and 103 from St. Lucia. Trial recruitment and retention have been previously detailed (33). Improved parenting knowledge scores and child cognitive development were the only reported benefits of the intervention (33).

The present study was an addendum to the parenting trial and is restricted to Jamaican families only. A subset of Jamaican mothers participating in the parenting trial was asked to give written informed consent to the collection of additional data when they attended the health clinic for their child’s routine 12 month clinic visit. At 72 months, families were again contacted using information collected on trial enrolment and appointments scheduled to conduct measurements. Data collected during trial enrolment was utilized along with data concerning maternal feeding styles collected at 12 months and child BMI obtained at age 18 and 72 months. Consent was obtained for each dyad prior to measurements at the study site. Approval for the study was obtained from the Ministry of Health and Wellness Jamaica and the University of the West Indies ethics committee.

### 2.2. Sample size

From the data available in Blake-Scarlett et al. (34) and Chang et al. (33), sample size calculation was performed to determine a 10% difference in BMI values at 72 months associated with feeding styles. With an intra cluster correlation of 0.03, a sample of 10 children per health clinic and design effect of 1.27 for clustered sampling, the number of families required to preserve 80% power at 5% level of statistical significance was 207.

### 2.3. Measurements

#### 2.3.1. Child characteristics

Baseline data on infant birthweight (kg) and gender were obtained from the children’s health records. Children were measured by trained staff using standardized protocols. Infant weight (kg) and recumbent length (cm) were obtained on trial enrolment and child age 18 months using a digital scale (Tanita BD-585 Digital Baby Scale) and length board (Seca 416 Infantometer Infant Measuring Board). These measurements were repeated at 72 months with weight measured using a digital scale (Seca 876, Class III) and height using a portable stadiometer (Seca 217 stadiometer). Weight and length/height measurements were recorded to the nearest 0.1 kg and 0.1 cm, respectively. Children’s clothing and shoes were removed before measurements.

Body Mass Index (BMI) (kg/m^2^) at age 18 and 72 months was calculated for each child using weight and length/height measurements. Age and gender specific BMI z-scores were generated from the World Health Organization (WHO) Anthro program (v.3.1.0) at both time points.

#### 2.3.2. Maternal sociodemographic characteristics

Maternal age (years), educational attainment, occupation, and depressive symptoms as well as household possessions, sanitation, water supply, crowding in the home were obtained on trial enrolment and at 72 months follow-up. Maternal depressive symptoms were assessed using the Center for Epidemiological Studies Depression Scale (CES-D) (35). All scale items were summed to give a total depression score with higher scores denoting higher levels of depressive symptoms. Maternal receptive vocabulary (verbal IQ) was measured using the Peabody Picture Vocabulary Test (PPVT-IV) on trial enrolment only (36). The PPVT-IV is comprised of a series of sets of four pictures per page that increase in difficulty. Mothers were asked to choose which picture best described the test word spoken by the examiner. The number of items correctly identified was summed to give a total receptive vocabulary score with higher scores indicating better receptive vocabulary. Both instruments have been previously modified for use in Jamaica.

Maternal weight (kg) and height (cm) were obtained at the infant’s 12 month clinic visit using a digital scale (Seca 876, Class III) and portable stadiometer (Seca 217 stadiometer) respectively, following standard procedures. Weight and height measurements were recorded to the nearest 0.1 kg and 0.1 cm, respectively. Maternal BMI (kg/m^2^) was calculated from these measurements and weight status classified as underweight (BMI <18.5 kg/m^2^), normal weight (BMI 18.5–24.9 kg/m^2^), overweight (BMI 25.0–29.9 kg/m^2^) and obesity (BMI ≥ 30.0 kg/m^2^) (37). The number of underweight mothers was small (*n* = 16) so women in the underweight and normal weight categories were combined. Maternal breastfeeding status at 12 months was also obtained and dichotomized into: (1) breastfeeding and (2) not breastfeeding.

##### 2.3.2.1. Maternal feeding styles

Maternal feeding styles were measured using the Toddler Feeding Behavior Questionnaire (TFBQ) (38). The questionnaire was designed and adapted from existing questionnaires and measures caregivers’ behavior and attitudes surrounding toddler feeding (24, 39, 40). Questionnaire items were reviewed and modified for cultural context by rewording some items to allow for better understanding and piloted with non-study mothers attending a health clinic with infants of similar age to the study group. Additional information on the reliability of the TFBQ has been described by Wright et al. (41). Confirmatory factor analysis was performed to identify the feeding styles using eigenvalues >1 and a five-factor solution, with an oblique rotation and standardized factor scores generated. Consistent with the original TFBQ the five toddler feeding subscales were: uninvolved, indulgent, restrictive, forceful and responsive feeding. Internal validity of the feeding subscales were high (Cronbach’s alpha (α) for uninvolved feeding = 0.79; indulgent feeding = 0.75; restrictive feeding = 0.73; forceful feeding = 0.70; responsive feeding = 0.70). The questionnaire was administered by trained interviewers to mothers visiting the health clinic for the child’s 12 month appointment.

### 2.4. Statistical analysis

STATA Statistical Program 12.0 (StataCorp, 2001) and SPSS Statistics for Windows 17.0 were used for all analyzes. Descriptive statistics were computed for maternal and child characteristics using means, standard deviations, frequencies and percentages. Principal component factor analysis using toilet facilities, water supply, household possessions and crowding in the home (number of persons per room) were used to derive an index of socioeconomic status (SES) with higher SES factor scores signifying better socioeconomic status. Wilcoxon rank sum (Mann–Whitney) test and Pearson’s chi-squared (*χ*^2^) test were used to compare feeding style scores and maternal and child characteristics of families with complete data with those with missing values. Student’s t-test was used to compare gender differences in feeding styles. Our analysis showed no differences in maternal feeding styles by gender so data were analyzed with gender pooled.

Multiple imputation using chained equation (MICE) was used to account for possible bias owing to missing data. Of the 239 families recruited for feeding style assessment, 155 (65%) had no missing data for any of the variables of interest, while 84 (35%) had 1 or more missing value(s). The proportions of missing values for the main variables to be used in the analysis are shown in Supplementary Table S1. A comparison of participants with complete data versus those with missing values showed minor differences (Table 1). Mothers with missing values were younger (*p* = 0.01) and had higher depressive symptoms scores (*p* = 0.01) than those with complete data. There were no other significant differences in maternal and child characteristics or feeding styles between participants with complete and missing data. For this reason, we determined the data to be missing at random (MAR) as the participants without and with missing data did not differ on most observed variables (42, 43). A stacked multiple imputed data set which consisted of 35 imputed data sets in addition to the original data were generated using STATA’s ‘mi’ commands. Based on the recommendation by White et al., 35 imputations were performed because 35% of the participants had any data missing (43). All the variables for inclusion in the analysis model (outcome and exposure variables) in addition to variables that were correlated with variables of interest and related to missingness were included in the imputation models. STATA’s ‘mi estimate’ command was used to create multivariable models using the stacked 35 multiple imputed data sets with estimates pooled by the software following Rubin’s rules.

**Table 1:** Comparison of maternal and child characteristics at enrolment for participants with complete data and those with missing data.

Characteristics	Complete data *n* = 155	Incomplete data *n* = 84	*P*-value
Maternal characteristics			
Age in years (mean ± SD)	26.01 ± 7.20	23.49 ± 6.71	0.01^*^
Vocabulary score (mean ± SD)	148.25 ± 25.75	141.36 ± 27.64	0.06
Depressive symptoms score (mean ± SD)	14.68 ± 10.50	18.58 ± 11.00	0.01^*^
SES factor score (mean ± SD)	−0.17 ± 0.99	−0.26 ± 0.92	0.48
Employment status, *n* (%)			
Never worked/unskilled	55 (35.5)	31 (36.9)	
Semi-skilled	71 (45.8)	37 (44.0)	0.97
Skilled or higher	29 (18.7)	16 (19.0)	
Weight status, *n* (%) †^ǂ^			
Underweight/normal	67(43.5)	44 (52.3)	
Overweight	34 (22.1)	23 (27.4)	0.15
Obesity	53 (34.4)	17 (20.2)	
Completed secondary school, *n* (%)	88 (56.8)	49 (58.3)	0.82
Breastfed at 12 months, *n* (%)ǂ	100 (64.5)	51 (60.7)	0.84
Child characteristics			
Birthweight (kg) (mean ± SD)	3.16 ± 0.39	3.14 ± 0.43	0.74

SD, Standard Deviation; SES, Socioeconomic status.

^†^*n* = 154 (numbers different due to missing data); Maternal weight categories according to the World Health Organization’s BMI criteria (Underweight, BMI z-score < 18.5; Normal weight, BMI z-score 18.5–24.9; Overweight, BMI z-score 25.0–29.9; Obesity, BMI z-score ≥ 30.0).

^ǂ^Maternal weight and breastfeeding status assessed at 12 months.

^*^p < 0.05 (differences using *χ*^2^ or Wilcoxon rank-sum test).

Multilevel linear regression models were used to explore whether maternal feeding styles at 12 months predicted child BMI z-scores at 72 months accounting for the cluster study design at the health clinic level and intervention group assignment. The covariates included in the models were selected based on previously published findings (41). Unadjusted regression models were initially explored then 2 adjusted models were generated–the first adjusted for child characteristics only (Model 1–gender, birthweight) and the second for dyad characteristics (Model 2–gender, birthweight; maternal BMI, maternal age at 72 months, receptive vocabulary score, depressive symptoms score at 72 months, breastfeeding status at 12 months and SES factor score at 72 months). Model adequacy was assessed by determining if there were any indications of heteroscedasticity for the variables in the analysis and whether the distribution of the standardized residuals deviated from the assumption of normality using a normal probability plot. There was no evidence of heteroscedasticity for the included variables. Results are presented as regression coefficients (β) and 95% confidence intervals (CI) with statistical significance defined as *p* < 0.05. Analyzes were performed for the complete case analysis (*n* = 155) and imputed data sets (*n* = 239).

To evaluate the indirect effects of feeding styles on child BMI z-scores at 72 months by 18 month BMI z-scores, the medsem STATA package for mediation analysis developed by Mehmetoglu was performed using 500 bootstraps (44). The medsem statistical package performs a mediational analysis based on a model estimated using STATA’s structural equation modeling (SEM) command and uses a modified Baron and Kenny 4 step mediation approach developed by Iacobucci et al. (45) and an alternative Zhao et al. bootstrap test of indirect effects approach (46). Mediation analysis was performed to determine whether the strength of the relationship between maternal feeding styles and 72 month BMI z-scores was significantly reduced by including BMI z-scores at 18 months into the path model. Indirect effect coefficients, standard errors, 95% confidence intervals and *p*-values for all model estimates were generated and used to determine the mediational role of 18 month BMI z-scores for each feeding style.

## 3. Results

Of the 396 mothers and infants enrolled in the parenting trial, 239 were recruited for feeding style assessment at 12 months. Of these participants, 155 had subsequent child anthropometric measurements at both 18 and 72 months. Multiple imputation was used to generate a complete dataset, as described in the analysis section.

### 3.1. Maternal and child characteristics

The comparison of maternal and child characteristics at trial enrolment for participants with complete data and those with missing values is shown in Table 1. At enrolment, mean maternal age was 26 years for mothers with complete data. Approximately 36% never worked or were in unskilled jobs. 56.8% of mothers had completed secondary/high school and 64.5% continued to breastfeed at 12 months. 22.1% were classified with overweight (BMI 25.0–29.9 kg/m^2^) and 34.4% with obesity (BMI ≥ 30 kg/m^2^). Compared to mothers with missing values, mothers with complete data were older (*p* = 0.01) and had lower mean depressive symptoms scores (*p* = 0.01; Table 1). There were no other significant differences in any of the maternal demographic characteristics at enrolment. In addition, child birthweight was not significantly different between the groups. Maternal characteristics measured at 72 months are described in Supplementary Table S2. Average maternal age was 32 years. Just over 16% of mothers never worked or were in unskilled jobs and approximately 64% completed secondary/high school.

Child characteristics measured at 18 and 72 months are shown in Supplementary Table S3. Mean ± SD BMI z-scores at 18 and 72 months were 0.26 ± 0.97 and 0.21 ± 1.16, respectively.

### 3.2. Feeding styles

There were no significant differences in restrictive (*p* = 0.10), forceful (*p* = 0.65), uninvolved (*p* = 0.28), indulgent (*p* = 0.43) and responsive (*p* = 0.28) feeding styles between participants with complete data (*n* = 155) and those with missing values (*n* = 84).

### 3.3. Maternal feeding styles as predictors of child BMI z-scores at 72 months

The multilevel regression analyzes examining restrictive feeding style at 12 months as a predictor of child BMI z-scores at 72 months for the imputed models is presented in Table 2. In the unadjusted models, maternal feeding restriction at 12 months was associated with higher BMI z-scores at 72 months [β (95%CI): 0.20 (0.02, 0.39)] and remained significant after controlling for both maternal and child covariates in Model 2 [β (95% CI): 0.19 (0.01, 0.37)]. Maternal BMI was the only covariate significantly associated with child BMI at 72 months with greater maternal BMI associated with increased child body size (Table 2). For the complete case analysis, restrictive feeding at 12 months predicted higher 72 month BMI z-scores in the unadjusted model [β (95%CI): 0.19 (0.02, 0.37)] and model adjusted for child covariates [β (95% CI): 0.20 (0.02, 0.38)] (Table not shown). However, after controlling for maternal covariates the association was no longer significant [β (95% CI): 0.17 (−0.01, 0.34)] (Table not shown). Uninvolved, forceful, indulgent and responsive feeding styles were not significant predictors of 72 month BMI z-scores for either complete case (Table not shown) or multiple imputation models (Supplementary Tables S4–S7).

**Table 2:** Multilevel linear regression analyzes between restrictive feeding at 12 months and BMI z-scores at age 72 months (*n* = 239).

	BMI z-scores at 72 months					
	Unadjusted		Model 1^ǂ^		Model 2^§^	
		*β* (95% CI)	*p*-value	*β* (95% CI)	*p*-value	*β* (95% CI)	*p*-value
Restrictive feeding†	0.20 (0.02, 0.39)	0.03^*^	0.21 (0.02, 0.39)	0.03^*^	0.19 (0.01, 0.37)	0.04^*^
Infant characteristics						
Gender	–	-	0.25 (−0.11, 0.60)	0.18	0.19 (−0.16, 0.54)	0.28
Birthweight	–	-	0.01 (−0.01, 0.04)	0.36	0.00 (−0.02, 0.03)	0.76
Maternal characteristics
Body mass index	–	–	–	–	0.05 (0.02, 0.07)	≤0.001^**^
Age	–	–	–	–	−0.01 (−0.04, 0.02)	0.48
Receptive vocabulary score	–	–	–	–	−0.00 (−0.01, 0.00)	0.39
SES factor score	–	–	–	–	0.13 (−0.01, 0.26)	0.07
Depressive symptoms score	–	–	–	–	−0.01 (−0.02, 0.01)	0.29
Breastfeeding status	–	–	–	–	0.05 (−0.29, 0.40)	0.77

CI, Confidence Interval. All models adjusted for clinic level clustering and intervention group assignment.

^†^Restrictive feeding style variable as standardized factor scores.

^ǂ^Adjusted for child gender and birth weight.

^§^Adjusted for child gender, birthweight, maternal BMI, maternal age at 72 months, maternal receptive vocabulary score, SES factor score at 72 months, maternal depressive symptoms score at 72 months, breastfeeding status at 12 months.

^*^p < 0.05; ^**^ *p* ≤ 0.001.

#### 3.3.1. 18 month BMI z-scores as a mediator between maternal feeding styles and child BMI z-scores at 72 months

**Figure 1:**
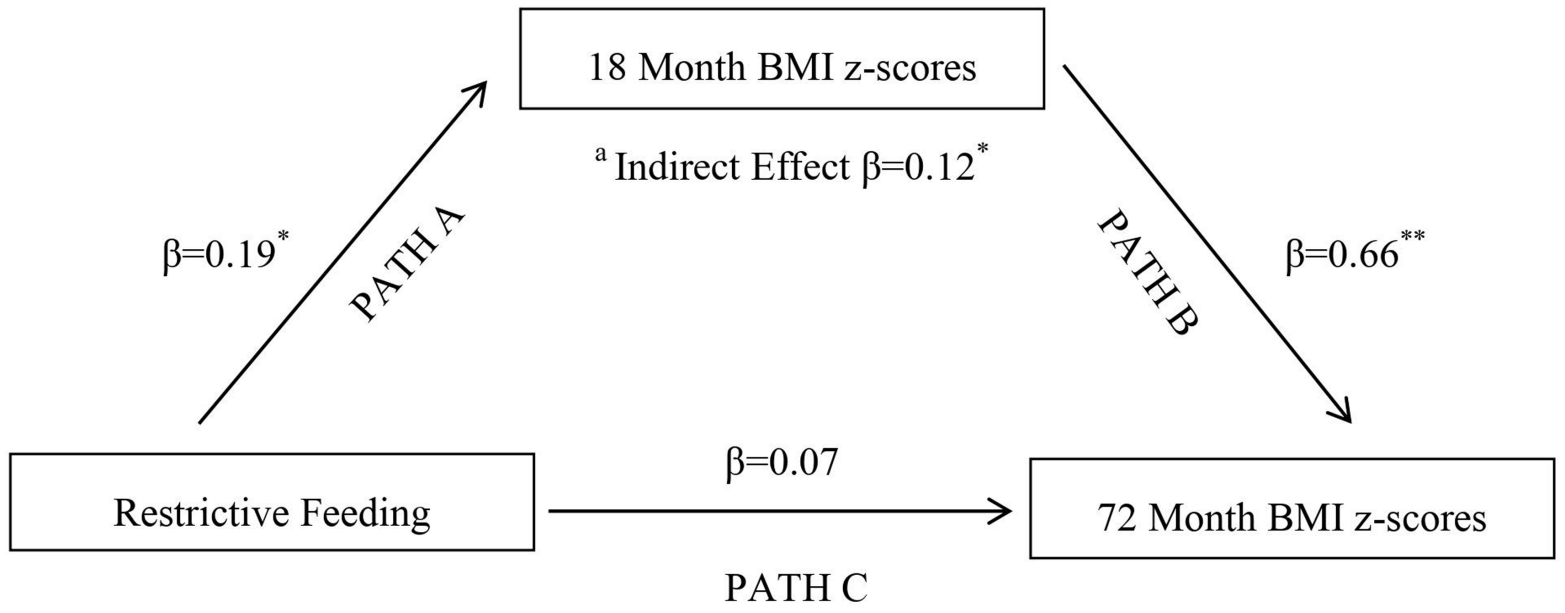
Multiple Imputation:18 month BMI Z-Scores Mediating Maternal Restrictive Feeding at 12 months and Child 72 month BMI Z-Scores. ^a^Indirect effect of restrictive feeding on child BMI z-scores at 72 months through the influence of 18 month BMI z-scores; ^*^p < 0.05, ^**^p < 0.001.

The direct path from restrictive feeding to child BMI z-scores at 72 months in the imputed dataset showed a non-significant association [β (95% CI): 0.07 (−0.09, 0.23); *p* = 0.39] (Table 3). Significant positive associations were observed for the direct paths from restrictive feeding to BMI z-scores at 18 months [β (95% CI): 0.19 (0.05, 0.32); *p* = 0.01] and BMI z-scores at18 months to 72 month BMI z-scores [β (95% CI): 0.66 (0.50, 0.82); *p* < 0.001] (Table 3). The Monte Carlo test confirmed the significance of an indirect effect of the relationship between restrictive feeding and child BMI z-scores at 72 months [β (95% CI): 0.12 (0.03, 0.22); *p* = 0.01] with 64% of the association mediated by 18 month BMI z-scores as illustrated in Figure 1. The path coefficients, standard errors (SE), confidence intervals and associated *p*-values for restrictive feeding models predicting child BMI z-scores at 72 months via BMI z-scores at 18 months are shown in Table 3.

**Table 3:** Bootstrapped path coefficients, standard errors, 95% confidence intervals and *p*-values for restrictive feeding models predicting 72 month BMI z-scores through BMI z-scores at 18 months (*n* = 239).

Model path^†^	β (95% CI)	SE	*p*–value
Direct effects			
(1) Restrictive feeding to child BMI z-scores at 72 months	0.07 (−0.09, 0.23)	0.08	0.39
(2) Restrictive feeding to child BMI z-scores at 18 months	0.19 (0.05, 0.32)	0.07	0.01^*^
(3) Child BMI z-scores at 18 months to child BMI z-scores at 72 months	0.66 (0.50, 0.82)	0.08	<0.001^**^
Indirect effect through child 18 month BMI z-scores			
Restrictive feeding to child BMI z-scores at 72 months	0.12 (0.03, 0.22)	0.05	0.01^*^

SE, Standard Error; BMI, Body Mass Index.

^†^All models adjusted for child gender, birth weight, maternal BMI, maternal age at 72 months, maternal receptive vocabulary score, SES factor score at 72 months, maternal depressive symptoms score at 72 months, breastfeeding status at 12 months and intervention group assignment.

^*^p < 0.05, ^**^p < 0.001.

Proportion of total effect that is mediated by 18 month BMI z-scores = (Indirect effect/Total effect) = (0.123/0.193) = 0.64. About 64% of the effect of restrictive feeding at 12 months on 72 month BMI z-scores is mediated by BMI z-scores at 18 months.

No significant associations were shown for indirect effects of uninvolved, forceful, indulgent and responsive feeding on 72 month BMI z-scores through 18 month BMI z-scores.

## 4. Discussion

In this study we found that there were longitudinal associations between restrictive feeding at age 12 months and child BMI z-scores at 72 months in a cohort of Jamaican mothers and children after controlling for maternal and child covariates. We also demonstrated that the association between maternal feeding restriction and child’s 72 month BMI z-scores was mediated through BMI z-scores at 18 months. Our investigation builds on the current literature regarding longitudinal associations between feeding styles and BMI z-scores among school aged children. This is important given the increasing prevalence of childhood overweight and obesity and the negative impacts of non-responsive feeding on child weight status.

Our observation complements the findings from Birch et al. (47, 48), Faith et al. (17, 49) and Johnson and Birch (50) that have documented associations of restrictive feeding with increased child BMI z-scores. Faith et al. reported that restrictive feeding at age 5 years was associated with increased BMI z-scores at age 7 years both with and without controlling for BMI z-scores at age 3 years (17) and Birch et al. observed that increased body adiposity among 5 year old girls was associated with feeding restriction (48). A review of the literature by Faith et al. reported parental feeding restriction as the only feeding domain associated with increased weight status and child eating (49). Feeding restriction may be used in an attempt to control weight gain by mothers with children at increased risk of overweight or obesity, either due to concern of the child’s future weight status or as a response to current weight (22, 25, 29). As such, mothers may use restrictive feeding to limit caloric intake and access to calorie dense foods (49). Restrictive feeding may initially reduce child weight but has been shown to exacerbate weight gain by increasing preference for restricted food and impeding the child’s ability to self-regulate energy intake in response to internal hunger and satiety cues (17, 28, 49, 51). Fisher et al. (28) and Jansen et al. (51) reported that restriction of energy dense foods such as snacks led to greater consumption by children once these restricted foods were available compared to unrestricted foods.

No significant associations were observed between uninvolved, forceful, indulgent and responsive feeding with 72 month child BMI z-scores (52, 53). In several prior cross-sectional studies, indulgent and uninvolved feeding were associated with higher child BMI in children 4–12 years (13, 14, 53–55), while responsive feeding was protective against higher child BMI (56, 57). Gubbels et al. reported lower child BMI with forceful feeding in Nordic mothers with children 5–7 years (18). Cross sectional designs may limit the inferences that can be deduced from the feeding style-BMI association.

BMI z-scores at 18 months mediated the association between restrictive feeding and 72 month BMI z-scores. Greater feeding restriction by parents with overweight children than parents with non-overweight children has been reported (17, 23, 28, 29, 48). Mothers may restrict food intake in children with overweight/obesity to prevent continued weight gain. Birch and Fisher’s path model showed that daughters with increased weight influenced greater maternal use of feeding restriction which perpetuated increased weight (23). Similar findings were reported by Francis et al. (29). These studies imply that restrictive feeding is at least partially influenced by the child’s weight (17, 23, 28, 29, 48). This supports the theory of a ‘child-responsive model’, presented by Webber et al. which proposed that a mother may alter their style of feeding in response to their child’s weight (25). The influence of BMI z-scores at 18 months on 72 month BMI z-scores could in part reflect children’s genetic predisposition to overweight/obesity (58, 59) in addition to rapid early growth related to diet and activity. Children at risk of overweight during infancy have been reported to be three times more likely to be overweight in later childhood than non-overweight children (58, 59).

There were strengths and limitations to the study. A validated questionnaire with good test retest reliability and high internal consistencies was used to assess maternal feeding styles. There were no significant feeding style differences between mothers with complete data and those with missing values. The longitudinal study design was another study strength and our investigation identified mediation by early childhood BMI z-scores in the relationship between restrictive feeding and later child BMI z-scores at 72 months. MICE was used to address missing data bias, due to sample attrition over the 6 year study period which limited the power for the complete case analysis. MICE preserved sample size and provided less biased estimates of the parameters. The generalizability of the findings to other ethnic groups and income levels are limited as families who participated were predominantly black and low/moderate-income. The retention bias in the sample of older, less depressed mothers may also affect generalizability. Another study limitation is the subjectivity of maternal report of feeding which may introduce bias and inaccuracy due to social desirability and misreporting. Although feeding styles was assessed using a validated questionnaire, other measures such as breastfeeding initiation, duration and exclusivity as well as additional measures of feeding styles such as direct observations of mealtime interactions would have added supplementary data and enhanced our results. Notwithstanding, direct observations of the feeding interaction may reduce bias but could also influence behaviors. The role and involvement of other caregivers during feeding and their use of feeding strategies which may influence child BMI z-scores were not investigated. It may be beneficial to investigate the influence of other caregivers in future studies.

Further research is needed to explore the longitudinal effects of feeding restriction among children affected by overweight risk compared to children who are not affected, as well as the variations in maternal motivation that may underlie restrictive feeding. There is some evidence that other factors such as parental concern about future weight (24), and parental perception of child size (24, 28, 58) may mediate the association between maternal feeding style and child BMI z-scores. Future studies could investigate the pathways by which these interact with feeding styles and its association with early and mid-childhood weight status.

## 5. Conclusion

In summary, our findings demonstrate the association of maternal feeding restriction at age 12 months with increased child BMI z-scores at 72 months in Jamaican mother–child pairs. No significant relationships were found between the other feeding styles and BMI z-scores at 72 months. An important addition to the body of knowledge is the demonstration of early childhood BMI z-scores as a mediator of the relationship between restrictive feeding and later child BMI z-scores with implications for overweight/obesity prevention and intervention programs as it emphasizes the importance of early overweight prevention. Additionally, our results suggest that the association between maternal feeding restriction and child BMI is bidirectional. Further prospective studies are needed to investigate additional mechanisms as well as bidirectional associations between feeding styles and increased body weight.

## Data availability statement

The raw data supporting the conclusions of this article will be made available by the authors, without undue reservation.

## Ethics statement

The studies involving human participants were reviewed and approved by the Ministry of Health and Wellness Jamaica and The University of the West Indies ethics committee. The participants provided their written informed consent to participate in this study.

## Author contributions

SC and SW lead the parenting trial in which this study was nested. AW, MT-R, and SW designed the research. AW and NG-D analyzed the data. AW wrote the paper and had primary responsibility for the final manuscript. SW, MT-R, NG-D, and SC provided the critical revisions and comments. All authors read and approved the final paper prior to submission.

## Funding

Funding for the reported research was provided by the Inter-American Development Bank (ATN/SF-12300-RG and RG-E1481) and the National Institutes of Health through Dr. Elsie Taveras and the Harvard Pilgrim group (NIH/HMS 5ULIRR025758).

## Conflict of interest

The authors declare that the research was conducted in the absence of any commercial or financial relationships that could be construed as a potential conflict of interest.

## Publisher’s note

All claims expressed in this article are solely those of the authors and do not necessarily represent those of their affiliated organizations, or those of the publisher, the editors and the reviewers. Any product that may be evaluated in this article, or claim that may be made by its manufacturer, is not guaranteed or endorsed by the publisher.

## Author disclaimer

The views expressed do not reflect those of the Inter-American Development Bank, its board of directors or the countries they represent.
