# Supplementary material for: Maternal feeding styles in infancy predict child body mass index z-scores at 72 months: findings from a cohort of Jamaican children
Source: Front Public Health. 2023 Jun 30;11:1161808. doi: 10.3389/fpubh.2023.1161808 (PMC10349535; doi:10.3389/fpubh.2023.1161808)
Supplement: Supplementary file 1 [file Data_Sheet_1.docx]

**Supplementary Table 1.** Number of Observed and Missing Data used in the Analysis

| **Main Variables** | **Number of Participants with Observed Data** | **Number of Participants with Missing Data** | **Percentage Missing Data (%)** |
| --- | --- | --- | --- |
| **Maternal Characteristics** |  |  |  |
| Vocabulary Score on Enrolment | 239 | 0 | 0 |
| Breastfed at 12 Months | 239 | 0 | 0 |
| Body Mass Index (BMI), kg/m^2^ | 238 | 1 | 0.42 |
| Uninvolved Feeding Factor Score | 239 | 0 | 0 |
| Restrictive Feeding Factor Score | 239 | 0 | 0 |
| Indulgent Feeding Factor Score | 239 | 0 | 0 |
| Forceful Feeding Factor Score | 239 | 0 | 0 |
| Responsive Feeding Factor Score | 239 | 0 | 0 |
| SES Factor Score at 72 Months | 176 | 63 | 26.36 |
| Depressive Symptoms Score at 72 Months | 176 | 63 | 26.36 |
| Age at 72 Months | 239 | 0 | 0 |
| **Child Characteristics** |  |  |  |
| Birthweight, kg | 239 | 0 | 0 |
| Gender | 239 | 0 | 0 |
| Intervention Group Assignment | 239 | 0 | 0 |
| 18 Month BMI z-score | 216 | 23 | 9.62 |
| 72 Month BMI z-score (*Outcome*) | 156 | 83 | 34.72 |

Abbreviations: BMI, Body Mass Index; SES, Socioeconomic Status

**Supplementary Table 2.** Maternal Characteristics assessed at 72 Month Follow-up

| **Characteristics** | |  |
| --- | --- | --- |
| Age in years (mean ± SD) | 32.01 ± 7.20 | |
| Depressive Symptoms Score (mean ± SD) | 19.48 ± 12.15 | |
| SES Factor Score (mean ± SD) | 0.03 ± 0.91 | |
| *Employment Status*, n (%) |  | |
| Never worked/Unskilled | 25 (16.1) | |
| Semi-Skilled | 78 (50.3) | |
| Skilled or Higher | 52 (33.5) | |
| *Completed Secondary School*, n (%) | 99 (63.9) | |

Abbreviations: SD, Standard Deviation; SES, Socioeconomic Status.

**Supplementary Table 3.** Child Characteristics at 18 and 72 Months Follow-up

| **Characteristics** | **18 Months** | **72 Months** |
| --- | --- | --- |
| *Gender male*, n (%) | 103 (48.1) | 72 (46.5) |
| Weight (kg) (mean ± SD) | 11.04 ± 1.36 | 21.87 ± 3.75 |
| BMI (kg/m^2^) (mean ± SD) | 11.05 ± 1.36 | 15.82 ± 1.98 |
| BMI z-scores (mean ± SD) | 0.26 ± 0.97 | 0.21 ± 1.16 |
| *Weight categories*, n (%) † |  |  |
| Underweight | 3 (1.9) | 4 (2.6) |
| Normal Weight | 120 (77.4) | 115 (74.2) |
| At Risk of Overweight | 32 (20.7) | 36 (23.3) |

Abbreviations: SD, Standard Deviation; BMI, Body Mass Index

^†^ Weight categories according to the World Health Organization’s BMI criteria (Underweight, BMI z-score < -2; Normal weight, BMI z-score ≥ -2 to 1; At risk of overweight, BMI z-score > 1)

**Supplementary Table 4.** Multilevel Linear Regression Analyses between Indulgent Feeding at 12 Months and BMI z-scores at Age

72 Months (*n=239*)

|  | **BMI z-scores at 72 Months** | | | | | |
| --- | --- | --- | --- | --- | --- | --- |
|  | **Unadjusted**  **β (95% CI) p-value** | | **Model 1^ǂ^**  **β (95% CI) p-value** | | **Model 2^§^**  **β (95% CI) p-value** | |
| Indulgent feeding† | -0.03 (-0.20, 0.14) | 0.71 | -0.03 (-0.20, 0.14) | 0.71 | -0.01 (-0.20, 0.17) | 0.88 |
| *Infant characteristics* |  |  |  |  |  |  |
| Gender | - | - | 0.23 (-0.13, 0.59) | 0.21 | 0.17 (-0.18, 0.53) | 0.34 |
| Birthweight | - | - | 0.01 (-0.01, 0.04) | 0.32 | 0.00 (-0.02, 0.03) | 0.73 |
| *Maternal characteristics* |  |  |  |  |  |  |
| Body mass index | - | - | - | - | 0.05 (0.02, 0.08) | ≤0.001^**^ |
| Age | - | - | - | - | -0.01 (-0.04, 0.01) | 0.37 |
| Receptive vocabulary score | - | - | - | - | -0.00 (-0.01, 0.00) | 0.22 |
| SES factor score | - | - | - | - | 0.14 (-0.00, 0.28) | 0.06 |
| Depressive symptoms score | - | - | - | - | -0.01 (-0.02, 0.01) | 0.41 |
| Breastfeeding status | - | - | - | - | 0.03 (-0.31, 0.38) | 0.86 |

Abbreviations: CI, Confidence Interval

All models adjusted for clinic level clustering and intervention group assignment.

^†^ Indulgent feeding style variable as standardized factor scores.

^ǂ^ Adjusted for child gender and birth weight.

^§^ Adjusted for child gender, birthweight, maternal BMI, maternal age at 72 months, maternal receptive vocabulary score, maternal depressive symptoms score at 72 months, breastfeeding status at 12 months, SES factor score at 72 months

^*^p< 0.05; ^**^ p≤0.001

**Supplementary Table 5.** Multilevel Linear Regression Analyses between Uninvolved Feeding at 12 Months and BMI z-scores at Age

72 Months (*n=239*)

|  | **BMI z-scores at 72 Months** | | | | | |
| --- | --- | --- | --- | --- | --- | --- |
|  | **Unadjusted**  **β (95% CI) p-value** | | **Model 1^ǂ^**  **β (95% CI) p-value** | | **Model 2^§^**  **β (95% CI) p-value** | |
| Uninvolved feeding† | 0.09 (-0.12, 0.30) | 0.42 | 0.10 (-0.11, 0.31) | 0.33 | 0.13 (-0.08, 0.34) | 0.24 |
| *Infant characteristics* |  |  |  |  |  |  |
| Gender | - | - | 0.24 (-0.12, 0.60) | 0.19 | 0.19 (-0.16, 0.55) | 0.28 |
| Birthweight | - | - | 0.02 (-0.01, 0.04) | 0.26 | 0.01 (-0.02, 0.03) | 0.62 |
| *Maternal characteristics* |  |  |  |  |  |  |
| Body mass index | - | - | - | - | 0.05 (0.02, 0.08) | ≤0.001^**^ |
| Age | - | - | - | - | -0.01 (-0.04, 0.02) | 0.41 |
| Receptive vocabulary score | - | - | - | - | -0.00 (-0.01, 0.00) | 0.35 |
| SES factor score | - | - | - | - | 0.14 (-0.01, 0.28) | 0.06 |
| Depressive symptoms score | - | - | - | - | -0.01 (-0.02, 0.01) | 0.37 |
| Breastfeeding status | - | - | - | - | 0.03 (-0.31, 0.38) | 0.86 |

Abbreviations: CI, Confidence Interval

All models adjusted for clinic level clustering and intervention group assignment.

^†^ Uninvolved feeding style variable as standardized factor scores.

^ǂ^ Adjusted for child gender and birth weight.

^§^ Adjusted for child gender, birthweight, maternal BMI, maternal age at 72 months, maternal receptive vocabulary score, maternal depressive symptoms score at 72 months, breastfeeding status at 12 months, SES factor score at 72 months

^*^p< 0.05; ^**^ p≤0.001

**Supplementary Table 6.** Multilevel Linear Regression Analyses between Forceful Feeding at 12 Months and BMI z-scores at Age 72

Months (*n=239*)

|  | **BMI z-scores at 72 Months** | | | | | |
| --- | --- | --- | --- | --- | --- | --- |
|  | **Unadjusted**  **β (95% CI) p-value** | | **Model 1^ǂ^**  **β (95% CI) p-value** | | **Model 2^§^**  **β (95% CI) p-value** | |
| Forceful feeding† | 0.08 (-0.09, 0.24) | 0.36 | 0.06 (-0.11, 0.23) | 0.49 | 0.04 (-0.13, 0.21) | 0.64 |
| *Infant characteristics* |  |  |  |  |  |  |
| Gender | - | - | 0.21 (-0.16, 0.57) | 0.26 | 0.16 (-0.19, 0.52) | 0.37 |
| Birthweight | - | - | 0.01 (-0.01, 0.04) | 0.34 | 0.00 (-0.02, 0.03) | 0.75 |
| *Maternal characteristics* |  |  |  |  |  |  |
| Body mass index | - | - | - | - | 0.05 (0.02, 0.07) | ≤0.001^**^ |
| Age | - | - | - | - | -0.01 (-0.04, 0.01) | 0.36 |
| Receptive vocabulary score | - | - | - | - | -0.00 (-0.01, 0.00) | 0.25 |
| SES factor score | - | - | - | - | 0.14 (-0.01, 0.27) | 0.06 |
| Depressive symptoms score | - | - | - | - | -0.01 (-0.02, 0.01) | 0.39 |
| Breastfeeding status | - | - | - | - | 0.04 (-0.31, 0.40) | 0.80 |

Abbreviations: CI, Confidence Interval

All models adjusted for clinic level clustering and intervention group assignment.

^†^ Forceful feeding style variable as standardized factor scores.

^ǂ^ Adjusted for child gender and birth weight.

^§^ Adjusted for child gender, birthweight, maternal BMI, maternal age at 72 months, maternal receptive vocabulary score, maternal depressive symptoms score at 72 months, breastfeeding status at 12 months, SES factor score at 72 months

^*^p< 0.05; ^**^ p≤0.001

**Supplementary Table 7.** Multilevel Linear Regression Analyses between Responsive Feeding at 12 Months and BMI z-scores at Age

72 Months (*n=239*)

|  | **BMI z-scores at 72 Months** | | | | | |
| --- | --- | --- | --- | --- | --- | --- |
|  | **Unadjusted**  **β (95% CI) p-value** | | **Model 1^ǂ^**  **β (95% CI) p-value** | | **Model 2^§^**  **β (95% CI) p-value** | |
| Responsive feeding† | 0.07 (-0.12, 0.27) | 0.46 | 0.08 (-0.12, 0.28) | 0.44 | 0.03 (-0.17, 0.23) | 0.77 |
| *Infant characteristics* |  |  |  |  |  |  |
| Gender | - | - | 0.21 (-0.15, 0.57) | 0.25 | 0.17 (-0.18, 0.52) | 0.35 |
| Birthweight | - | - | 0.02 (-0.01, 0.05) | 0.25 | 0.01 (-0.02, 0.03) | 0.68 |
| *Maternal characteristics* |  |  |  |  |  |  |
| Body mass index | - | - | - | - | 0.05 (-0.02, 0.07) | ≤0.001^**^ |
| Age | - | - | - | - | -0.01 (-0.04, 0.01) | 0.37 |
| Receptive vocabulary score | - | - | - | - | -0.00 (-0.01, 0.00) | 0.26 |
| SES factor score | - | - | - | - | 0.14 (-0.01, 0.28) | 0.06 |
| Depressive symptoms score | - | - | - | - | -0.01 (-0.02, 0.01) | 0.46 |
| Breastfeeding status | - | - | - | - | 0.03 (-0.32, 0.38) | 0.86 |

Abbreviations: CI, Confidence Interval

All models adjusted for clinic level clustering and intervention group assignment.

^†^ Responsive feeding style variable as standardized factor scores.

^ǂ^ Adjusted for child gender and birth weight.

^§^ Adjusted for child gender, birthweight, maternal BMI, maternal age at 72 months, maternal receptive vocabulary score, maternal depressive symptoms score at 72 months, breastfeeding status at 12 months, SES factor score at 72 months

^*^p< 0.05; ^**^ p≤0.001
